# Supplementary material for: Age-Related Changes in Predictors of BMI in 6, 9 and 12-Year-Old Boys and Girls: The NW-CHILD Study
Source: J Funct Morphol Kinesiol. 2025 Aug 18;10(3):320. doi: 10.3390/jfmk10030320 (PMC12372163; doi:10.3390/jfmk10030320)
Supplement: Supplementary file 1 [file jfmk-10-00320-s001.zip › jfmk-3699484-supplementary.pdf]

Collinearity analysis without mass and stature

Condition Index (CI) Thresholds

- CI < 10 → no concern
- CI 10–30 → **moderate collinearity**
- CI > 30 → **severe collinearity** concern

In your output:

- Dimensions 5 (CI = 18.204), 6 (CI = 26.595), and especially 7 (CI = 55.337) **indicate moderate to severe collinearity**.

Collinearity Diagnostics

Model	Dimension	Eigenvalue	Condition Index	Variance Proportions						
				(Constant)	Sub-scap 2010	Triceps 2010	Calf 2010	Mid-circ 2010	Fat % 2010	Muscle % 2010
1	1	6,678	1,000	,00	,00	,00	,00	,00	,00	,00
	2	,181	6,072	,01	,05	,01	,03	,00	,01	,02
	3	,065	10,140	,00	,00	,03	,11	,00	,66	,01
	4	,044	12,282	,00	,63	,01	,24	,00	,14	,00
	5	,020	18,204	,01	,10	,78	,62	,00	,00	,02
	6	,009	26,595	,17	,13	,15	,00	,02	,06	,88
	7	,002	55,337	,81	,09	,02	,00	,98	,11	,08

a. Dependent Variable: BMI 2010

PCA analysis T1

Descriptive Statistics

	Mean	Std. Deviation	Analysis N
Sub-scap 2010	6,8261	3,07811	332
Triceps 2010	8,9134	3,58236	332
Calf 2010	8,782	3,7848	332
Mid-circ 2010	54,563	5,4809	332
Fat % 2010	17,708	7,0322	332
Muscle % 2010	25,955	3,8650	332

KMO and Bartlett's Test

Kaiser-Meyer-Olkin Measure of Sampling Adequacy.		,818
Bartlett's Test of Sphericity	Approx. Chi-Square	1412,939
	df	15
	Sig.	<.001

Anti-image Matrices

		Sub-scap 2010	Triceps 2010	Calf 2010	Mid-circ 2010	Fat % 2010	Muscle % 2010
Anti-image Covariance	Sub-scap 2010	,275	-,094	-,031	-,096	-,031	,144
	Triceps 2010	-,094	,187	-,125	-,025	-,031	-,074
	Calf 2010	-,031	-,125	,265	-,006	-,039	-,038
	Mid-circ 2010	-,096	-,025	-,006	,318	-,142	-,190
	Fat % 2010	-,031	-,031	-,039	-,142	,451	,146
	Muscle % 2010	,144	-,074	-,038	-,190	,146	,671
Anti-image Correlation	Sub-scap 2010	,836[a]	-,414	-,117	-,326	-,089	,336
	Triceps 2010	-,414	,812[a]	-,564	-,103	-,108	-,209
	Calf 2010	-,117	-,564	,859[a]	-,020	-,112	-,090

	Mid-circ 2010	-,326	-,103	-,020	,829[a]	-,375	-,412
	Fat % 2010	-,089	-,108	-,112	-,375	,870[a]	,265
	Muscle % 2010	,336	-,209	-,090	-,412	,265	,481[a]

a. Measures of Sampling Adequacy(MSA)

Communalities

	Initial	Extraction
Sub-scap 2010	1,000	,822
Triceps 2010	1,000	,859
Calf 2010	1,000	,786
Mid-circ 2010	1,000	,775
Fat % 2010	1,000	,714
Muscle % 2010	1,000	,969

Extraction Method: Principal Component Analysis.

Covariance Matrix

	Sub-scap 2010	Triceps 2010	Calf 2010	Mid-circ 2010	Fat % 2010	Muscle % 2010
Sub-scap 2010	9,475	8,807	8,456	11,977	14,015	1,329
Triceps 2010	8,807	12,833	11,520	14,280	16,048	4,548
Calf 2010	8,456	11,520	14,325	13,844	16,074	4,437
Mid-circ 2010	11,977	14,280	13,844	30,041	25,503	8,391
Fat % 2010	14,015	16,048	16,074	25,503	49,452	1,959
Muscle % 2010	1,329	4,548	4,437	8,391	1,959	14,938

Total Variance Explained

Component	Initial Eigenvalues			Extraction Sums of Squared Loadings			Rotation Sums of Squared Loadings		
	Total	% of Variance	Cumulative %	Total	% of Variance	Cumulative %	Total	% of Variance	Cumulative %
1	3,922	65,364	65,364	3,922	65,364	65,364	3,702	61,699	61,699
2	1,003	16,722	82,086	1,003	16,722	82,086	1,223	20,387	82,086
3	,448	7,465	89,551						
4	,302	5,028	94,579						
5	,195	3,256	97,835						
6	,130	2,165	100,000						

Extraction Method: Principal Component Analysis.

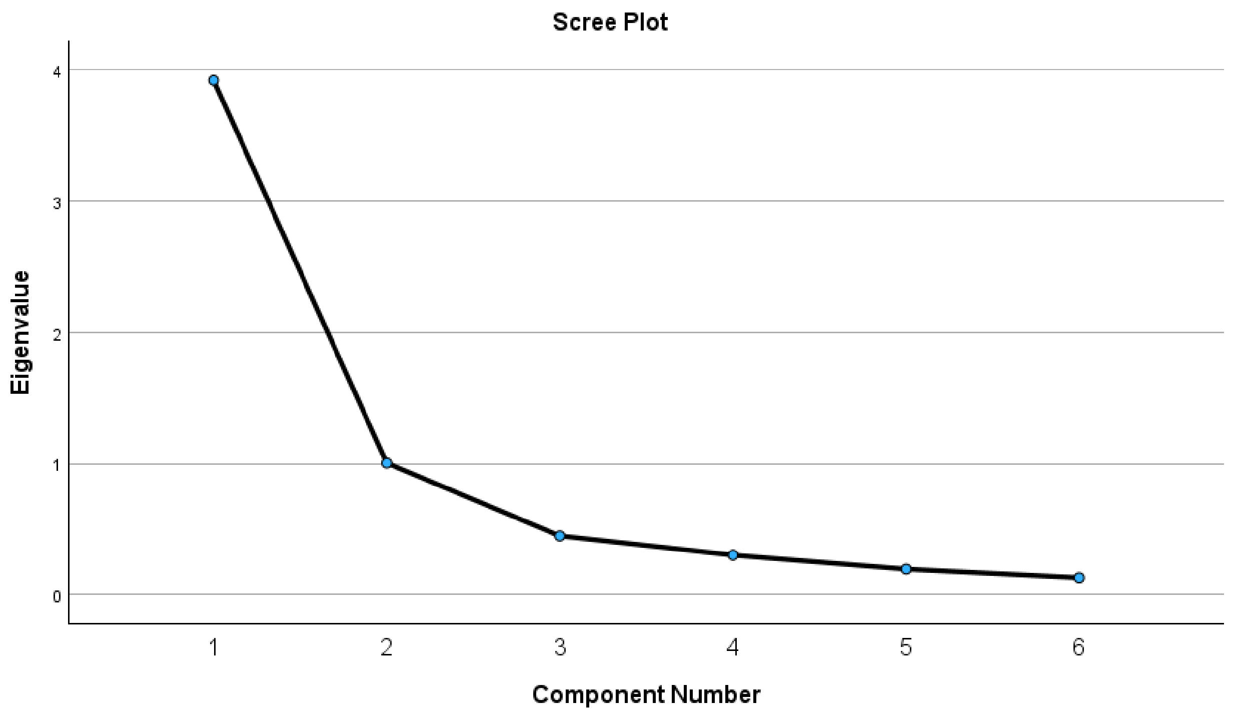

Component Matrix[a]

	Component	
	1	2
Sub-scap 2010	,874	-,242
Triceps 2010	,926	,013
Calf 2010	,886	,011
Mid-circ 2010	,873	,114
Fat % 2010	,786	-,311
Muscle % 2010	,367	,913

Extraction Method: Principal Component Analysis.

a. 2 components extracted.

Rotated Component Matrix[a]

	Component	
	1	2
Sub-scap 2010	,907	,007
Triceps 2010	,887	,267
Calf 2010	,849	,254
Mid-circ 2010	,808	,349
Fat % 2010	,841	-,084
Muscle % 2010	,102	,979

Extraction Method: Principal Component Analysis.

Rotation Method: Varimax with Kaiser Normalization.

a. Rotation converged in 3 iterations.

Component Transformation Matrix

Component	1	2
1	,962	,274
2	-,274	,962

Extraction Method: Principal Component Analysis.

Rotation Method: Varimax with Kaiser Normalization.
